# The effect of surgeon’s preference for hybrid or cemented fixation on the long-term survivorship of total knee replacement: An analysis of 39,623 prostheses from the Australian Orthopaedic Association National Joint Replacement Registry

**DOI:** 10.1080/17453674.2018.1449466

**Published:** 2018-03-12

**Authors:** Christopher J Vertullo, Stephen E Graves, Yi Peng, Peter L Lewis

**Affiliations:** 1 Knee Research Australia, Gold Coast, Australia; 2 Gold Coast Orthopaedic Research and Educational Alliance, Menzies Health Institute, Griffith University, Gold Coast, Australia; 3 Australian Orthopaedic Association National Joint Replacement Registry, SAHMRI, Adelaide, Australia

## Abstract

**Background and purpose:**

Recent direct comparative reports suggest that hybrid fixation may have a similar or superior outcome to cemented fixation in total knee replacement (TKR); however, a paucity of long-term data exists. To minimize the confounders of a direct comparison, we performed an instrumental variable analysis examining the revision rate of 2 cohorts of patients based on their surgeon’s preference for cemented or hybrid fixation.

**Methods:**

Registry data were obtained from 1999 until 2015 for 2 cohorts of patients who received minimally stabilized TKR, defined as those treated by high-volume hybrid fixation preferring surgeons, designated routinely hybrid (RH), and those treated by high-volume cemented fixation preferring surgeons, designated routinely cemented (RC).

**Results:**

At 13 years, the cumulative percentage revision of the RC cohort was 4.8% (CI 4.1–5.7) compared with 5.5% (CI 3.5–8.7) for the RH cohort. The revision risk for each cohort was the same for all causes (HR =1.0 (CI (0.84–1.20)), non-infective causes, and for infection. This finding was irrespective of patient age or sex, patella resurfacing, and with non-cross-linked polyethylene (NXLPE). The RH cohort who received cross-linked polyethylene (XLPE) had a lower revision risk than the RC cohort with XLPE (HR =0.57 (0.37–0.88), p = 0.01).

**Interpretation:**

The risk of revision for the patients of surgeons who prefer cemented fixation in minimally stabilized TKR is the same as for the patients of surgeons who prefer hybrid fixation, except when used with XLPE, where hybrid fixation has a lower revision risk.

The optimum fixation in total knee replacement (TKR) is controversial, with cemented fixation remaining the most common method internationally (National Joint Registry, AOA National Joint Registry 2016), compared with hybrid fixation (cemented tibia and cementless femur) or cementless fixation of both components. Hybrid fixation was introduced to overcome the perceived concerns over cementless fixation of the tibia while attempting to minimize femoral bone loss, decrease operative time, and reduce the polymethylmethacrylate burden of the joint (Wright et al. 1990, Kraay et al. 1991, Faris et al. 2008).

While recent reports suggest that hybrid fixation may have a similar or superior outcome to cemented fixation (Petursson et al. 2015), a paucity of long-term data exists concerning this method of fixation in TKR (Nakama et al. 2012). While cemented fixation of both components has excellent long-term survivorship in national registries (National Joint Registry, AOA National Joint Registry 2016), in the Australian Orthopaedic Association National Joint Replacement Registry (AOANJRR), hybrid fixation has the lowest revision risk overall when compared with cemented and cementless TKR. However, this revision risk is altered when prosthesis stability is considered. In posterior stabilized (PS) TKR, cemented fixation has the lowest revision risk. Conversely, in minimally stabilized (MS) TKR there is no difference between hybrid fixation and cemented fixation, and both have a lower revision risk compared with cementless fixation. The reasons why hybrid fixation has a lower risk for all prosthesis types, but not when PS or MS TKR are considered individually, are uncertain.

Hybrid fixation may not be appropriate for all patients, particularly in patients with osteoporosis, osteonecrosis, complex deformity, rheumatoid arthritis, or inaccurate bone resection (Scott 2012). These factors may bias registry data against cemented fixation when directly compared. Conversely, hybrid fixation may be used more commonly in younger active patients, which may bias registry data against hybrid fixation.

Previous registry studies have performed direct comparison of hybrid versus cemented TKR survivorship rates. In contradistinction, we performed an instrumental variable analysis based on surgeon preference for different prosthesis fixation options rather than the actual prosthesis received. This technique compares the revision rate of all primary minimally stabilized TKR undertaken by high-volume surgeons who preferred hybrid fixation TKR to those undertaken by high-volume surgeons who preferred cemented TKR. The rationale for this instrumental variable approach is that it has the capacity to remove the confounding by indication or disease severity between hybrid and cemented fixation that is not possible by directly comparing hybrid and cemented TKR implant registry revision rates (Vertullo et al. 2017).

Our primary hypothesis was that there would be no difference in the revision rate when the 2 patient cohorts were compared. Our secondary hypothesis was that there would be no difference in the revision rate with sub-analysis based on age, sex, type of polyethylene, and patella resurfacing.

## Methods

### Study design

2 groups of surgeons, who performed more than 50 TKR per year, differing by their femoral fixation preference were selected to perform an instrumental variable survivorship analysis (Newhouse and McClellan 1998; Stukel et al. 2007 references missing) with surgeon preference serving as the instrument, using data from the Australian Orthopaedic Association National Joint Replacement Registry (AOANJRR). A revealed preference for a femoral fixation option was defined as choosing to utilize it greater than 90% of the time, based on prior studies relating to knee implant choice by surgeons (Vertullo et al. 2017). Hence, a hybrid preferring (HP) surgeon used hybrid fixation at least 90% of the time, and the patient cohort treated by those surgeons has been termed routinely hybrid (RH). A cemented preferring (CP) surgeon used cemented fixation at least 90% of the time and the patient cohort treated by those surgeons has been termed routinely cemented (RC).

This study included all MS primary TKR undertaken for osteoarthritis (OA) with fixed cemented tibial components and cemented or cementless femoral components, undertaken by the 2 groups of surgeons and reported to the registry, irrespective of patella resurfacing. PS TKR, mobile bearing TKR, cementless (cementless femur and tibia), reverse hybrid (cemented femur and cementless tibia) TKR, and TKR with a higher than anticipated risk of revision in the AOANJRR were excluded, as were non-osteoarthritic patients such as those with rheumatoid arthritis, or osteonecrosis (AOA National Joint Registry 2016). Data for the 2 patient cohorts and their treating surgeons were obtained from the AOANJRR from September 1, 1999, until December 31, 2015. The AOANJRR commenced data collection in 1999 and includes data on more than 98% of arthroplasty procedures performed nationally since 2002 (AOA National Joint Registry 2016). The AOANJRR collects information on prosthesis type by catalogue and lot number, as well as cement used for each component by catalogue and lot number. Intended component fixation method is confirmed by linking component data to an internally developed comprehensive international prostheses library, validated with both manufacturers and other registries. If the actual component fixation method is not recorded at time of surgery (approximately less than 1% of TKR), the absent information is then obtained from the hospital. This linking of actual and intended fixation ensures almost complete accuracy in determining and verifying the fixation used in every procedure.

The AOANJRR defines MS prostheses as those that have a flat or dished tibial articulation regardless of congruency, hence this group includes cruciate retaining and ultracongruent polyethylene options. PS prostheses provide additional posterior stability, most commonly using a peg and box design. Cross-linked-polyethylene (XLPE) was defined as ultra-high-molecular-weight polyethylene that has been irradiated with high-dose (≥ 50 kGy) radiation, regardless of re-melting or annealing (de Steiger et al. 2015).

Time to first revision was the principal outcome measure, with revision being defined as any procedure that involves the insertion, removal, and/or replacement of a prosthesis. Reasons for revision and the type of revision were reported for procedures undertaken by both groups of surgeons. Further analyses based on patient’s age, sex, patella resurfacing, and the type of polyethylene were also undertaken. Analysis of surgeon practice public/private mix, years of contribution to registry, number of TKR in registry, and hospital arthroplasty volume was also undertaken.

### Statistics

Kaplan–Meier estimates of survivorship were used to estimate the time to the first revision, with right censoring for death or closure of the database at the time of analysis. The unadjusted cumulative percentage revision (CPR) of the primary arthroplasty, along with 95% confidence intervals (CI), was calculated using unadjusted point-wise Greenwood estimates. Hazard ratios (HR), calculated using Cox proportional hazard models and adjusted for age and sex, were used to make statistical comparisons of the rate of revision between the 2 cohorts. All tests were 2-tailed at the 5% level of significance. The analysis was performed using SAS version 9.3 (SAS Institute Inc., Cary, NC, USA).

### Ethics, funding, and potential conflicts of interest

The AOANJRR is approved by the Australian Federal Government as a Declaration of Quality Assurance Activity under section 124X of the Australian Federal Health Insurance Act, 1973. All investigations were conducted in accordance with ethical principles of research (the Helsinki Declaration II). No funding was received specific to this study and there are no competing interests to declare.

## Results

**Figure 1.**
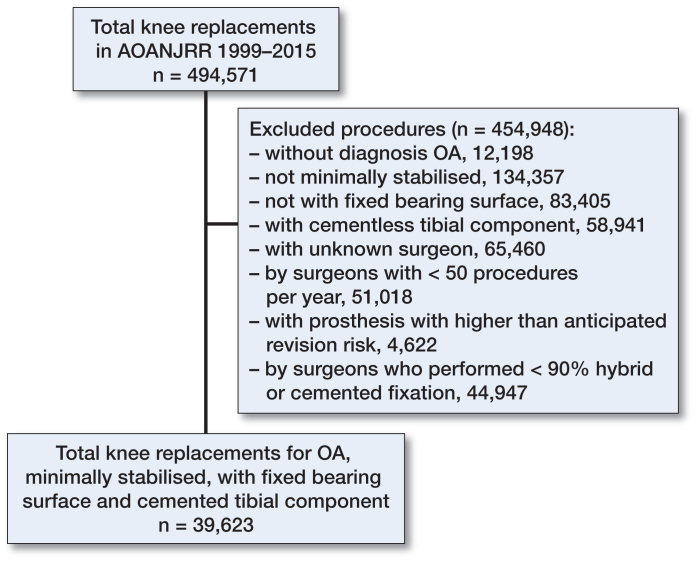
Flow diagram of total knee replacement exclusions.

There were 39,623 primary TKR that met the inclusion criteria, undertaken by 108 surgeons, with 30,544 cemented TKRs and 9,079 hybrid TKRs (Figure 1). Most surgeons were cemented preferring (87%) and they undertook 77% of the included procedures. The hybrid preferring surgeons each performed on average more of the included TKR compared with the CP surgeons, with a mean of 649 TKR/surgeon compared with a mean of 325 TKR/surgeon, respectively (Table 1). The CP surgeons undertook hybrid fixation in 0.7% of their TKR and the HP surgeons undertook cemented fixation in 2.8% of their TKR.

**Table 1.** Total knee replacements (TKRs) included in the analysis by surgeon fixation preference

	Surgeon preference		
	Cemented fixation	Hybrid fixation	Total
No. of surgeons	94	14	108
TKR/surgeon, mean (SD)	325 (359)	649 (374)	
interquartile range	[25–513]	[311–903]	
Type of TKR, n (%)			
Cemented	30,318 (99.3)	256 (2.8)	30,574
Hybrid	226 (0.7)	8,823 (97.2)	9,049
Total	30,544 (100)	9,079 (100)	

The demographics of each surgeon group had some differences, with the proportion of surgeons who worked in both private and public settings being higher in the HP surgeons (93%) compared with the CP surgeons (70%). The CP surgeons had contributed to the registry for more years (mean 9.6 years) compared with the HP surgeons (mean 6.2 years). Otherwise, the mean number of TKR in the registry, volume of all TKR/year and respective hospital arthroplasty volume was comparable for each group (Table 2).

**Table 2.** Surgeon practice data

	Surgeon preference	
	Cemented fixation	Hybrid fixation
Type of practice:		
Public and private/private only/public only (%)	70/25/5	93/7/0
Surgeons’ time in the registry **^a^** (year)	6.2 (4.9) [2.1–7.8]	9.6 (4.3) [6.7–12.7]
No. of total knee replacements in registry **^a^**	805 (435) [503–1040]	781 (337) [581–987])
Included and excluded total knee replacements per year **^a^**	96 (41) [68–109]	85 (22) [66–106])
Hospital volume per year of all lower-limb arthroplasties **^a^**	501 (373) [235–618]	616 (435) [276–840]

a The values are given as the mean (SD) [interquartile range].

**Figure 2.**
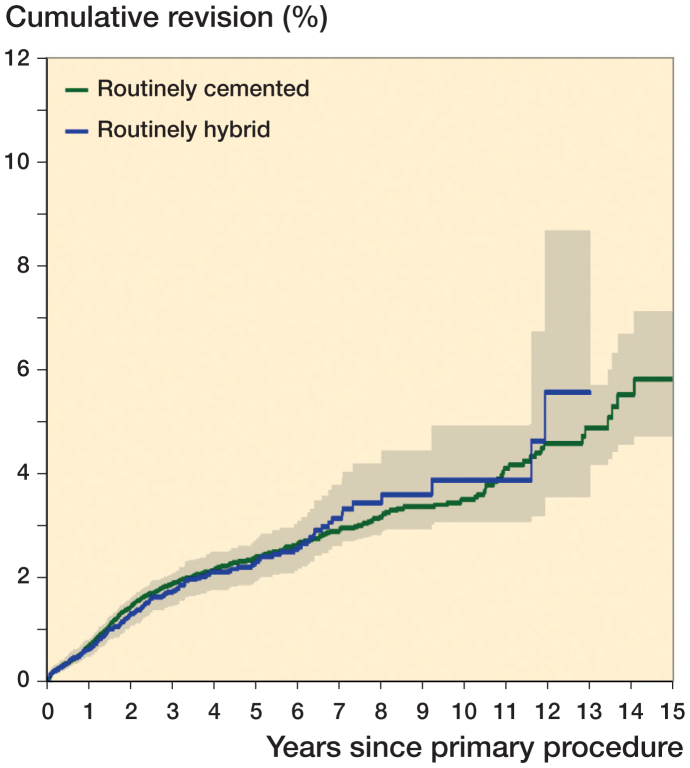
Cumulative percentage revision of primary total knee replacement by surgeon fixation preference in patients with osteoarthritis. Gray zones represent 95% confidence intervals. HR adjusted for age and sex: Routinely cemented (RC) versus routinely hybrid (RH), entire period: HR =1.00 (0.84–1.20).

Both patient cohorts had similar mean age and gender demographics (Table 3), except that a greater proportion of the routinely hybrid cohort were undertaken at public hospitals (77%) compared with the routinely cemented cohort (33%). At 13 years, the CPR of the RC cohort was 4.8% (CI 4.1–5.7) compared with 5.5% (CI 3.5–8.7) for the RH cohort. The revision risk for each cohort was the same for all causes (HR =1.0 (CI (0.84–1.20)) (Figure 2), non-infective causes (HR =1.0 (0.81–1.24)), and for infection (HR =1.0 (0.73–1.38)).

Number at risk (Figure 2)

**Table:** 

Year	RC	RH
0	30,544	9,079
1	25,107	7,422
2	20,336	5,948
3	16,260	4,596
4	12,755	3,378
5	9,541	2,461
6	6,901	1,759
7	4,594	1,055
8	3,021	577
9	2,421	383
10	1,919	254
11	1,433	165
12	1,009	100
13	618	59
14	335	31
15	127	9

**Table 3.** Demographic characteristics of the study cohorts

	Routinely cemented	Routinely hybrid
	n = 30,544	n = 9,079
Female (%)	57	58
Age, mean (SD)	68.8 (9.0)	69.0 (9.4)
Male (%)	43	42
Age, mean (SD)	68.4 (8.8)	68.7 (9.0)
Type of hospital (%)		
Public	33	77
Private	67	23

**Figure 3.**
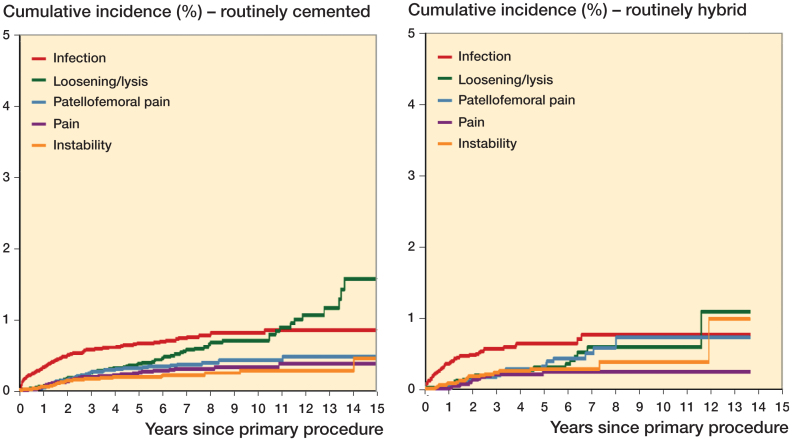
Cumulative incidence revision diagnosis of primary total knee replacement by surgeon fixation preference in patients with osteoarthritis.

The 5 most common diagnoses at revision were similar in each cohort (Figure 3) (Table 4, see Supplementary data). The types of revision were similar between the cohorts.

When the effects of age were examined, the revision risk for the RC cohort who were less than 65 years was similar to the RH cohort who were less than 65 years (HR =1.1 (1 = 0.83–1.42)). Similarly, the revision risk for patients older than 65 years (HR =0.96 (0.76–1.21)) was similar between the 2 cohorts.

Stratification of patients into males and females aged less than 65 years (Figure 4, see Supplementary data) and greater than 65 years revealed the same revision risk both for males in each cohort, and for females in each cohort.

**Figure 5.**
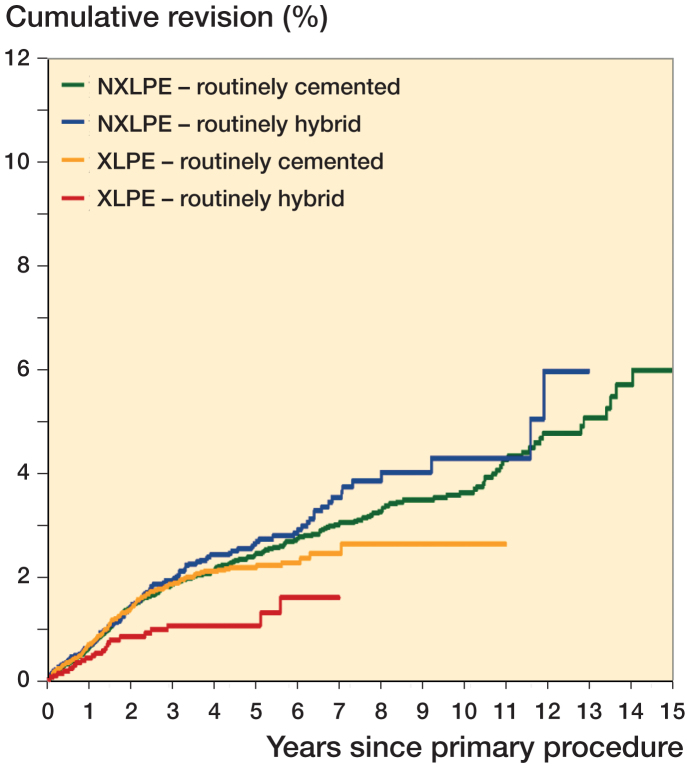
Cumulative percentage revision of primary total knee replacement by polyethylene type and surgeon fixation preference in patients with osteoarthritis. HR adjusted for age and sex, entire period: NXLPE RC vs NXLPE RH: HR =0.89 (0.73–1.08), p = 0.2 XLPE RH vs NXLPE RH: HR =0.48 (0.31–0.75, p = 0.001 NXLPE RC vs XLPE RC: HR =0.94 (0.79–1.12), p = 0.5 XLPE RH vs XLPE RC: HR =0.57 (0.37–0.88), p = 0.01

XLPE usage was more common in the RC cohort (47%) than in the RH cohort (29%). When the effects of XLPE were examined, the RH cohort with XLPE had a lower revision risk than the RC cohort with XLPE (HR =0.57 (0.37–0.88)) (Figure 5). Revision risk with non-cross-linked-polyethylene (NXLPE) was the same between the cohorts.

Patella resurfacing was more common in the RC cohort (61%) compared with the RH cohort (51%). When the effects of patella resurfacing were examined, both cohorts had the same revision risk with and without patella resurfacing (Figure 6, see Supplementary data).

**Figure 7.**
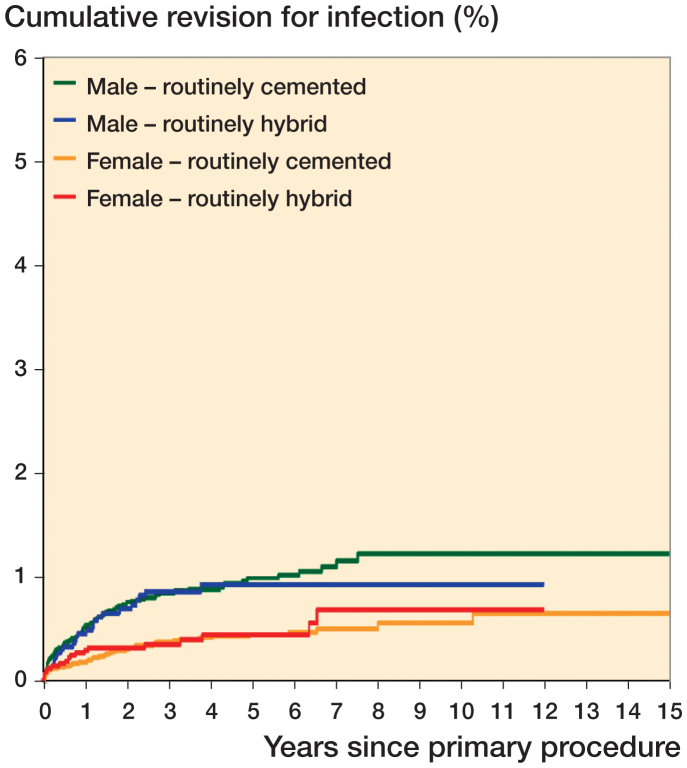
Cumulative percentage revision for infection of primary total knee replacement by patient gender and surgeon fixation preference in patients with osteoarthritis. HR adjusted for age, entire period: Male RC vs male RH: HR =1.07 (0.71–1.63), p = 0.7 Male RH vs female RH: HR =1.90 (1.07–3.37, p = 0.03 Male RC vs female RC: HR =2.26 (1.65–3.09), p < 0.001 Female RC vs female RH: HR =0.90 (0.54–1.49), p = 0.7

When the revisions for infection in each cohort were stratified by sex there was no difference between males and females in each cohort (Figure 7).

## Discussion

The advantages and disadvantages of cement fixation have been debated for decades; however, the choice of TKR fixation typically remains the preference of the surgeon, characteristically founded in efforts to maximize the long-term outcomes of their patients (Kobs and Lachiewicz 1993), with loosening and lysis remaining the dominant reasons for revision.

In this analysis, patients of surgeons who preferred hybrid fixation had the same long-term risk of revision compared with the patients of those surgeons who preferred cemented fixation. This finding was irrespective of patient’s age or sex, and whether the patella was resurfaced or not. When reasons for revision were stratified into non-infective and infective, there was no difference between the 2 cohorts overall. There was also no difference between the 2 cohorts for those who received NXLPE, but the patients of surgeons who preferred hybrid fixation and received XLPE had a lower revision risk than the patients of surgeons who preferred cemented fixation and received XLPE.

Cement disease (Jones and Hungerford 1987) was described in 1987, suggesting particles of polymethylmethacrylate were the primary cause of osteoclast-induced failure at the prosthesis–bone interface. More recently, the theory of cement disease has been discarded in favor of wear-particle induced lysis and loosening, which primarily focuses on bearing surface generated particles rather than those from the fixation interface (Harris 1994). Furthermore, cement has been suggested as a possible protective barrier, or seal, to wear particle-laden synovial fluid ingress into the prosthesis¬–bone interface (Harris et al. 1996). Our results are not in keeping with either the cement disease theory or cement as a seal theory.

Despite a paucity of supporting clinical data, cementless fixation of the femur and tibia has been recommended as the optimum biologic TKR fixation solution for younger at-risk patients when compared with cemented fixation (Dorr 2002). However, in registry studies, the long-term revision risk of cementless fixation is higher than cemented and hybrid fixation and in smaller clinical series outcomes of cementless fixation of the femur and tibia remain similar, or inferior to, cemented fixation of the femur and tibia (Pulido et al. 2015, Dalury 2016). It is for this reason we did not examine cementless fixation of both components in this analysis. Cementless fixation of the tibia does not reduce the revision risk or migration when compared with cemented fixation in radiostereometric trials (Carlsson et al. 2005), in registry studies (Graves et al. 2016), or clinical series (Behery et al. 2016). Given that the tibia remains the component most at risk for failure (Voigt and Mosier 2011), hybrid fixation was introduced as a pragmatic alternative to employ the advantages of 2 differing fixation philosophies (Kraay et al. 1991, Petursson et al. 2015).

Hybrid fixation has similar outcomes to cemented fixation in direct comparisons (Pelt et al. 2013), clinical series (Choi et al. 2012, McLaughlin and Lee 2014), and in some registry reports, a lower revision risk (Petursson et al. 2015, AOA National Joint Registry 2016). Petursson et al. (2015) performed a registry review of 3 different fixed and mobile bearing TKR designs, reporting that 1 of 3 three designs examined had a lower revision risk in the hybrid version. When this prosthesis, mainly performed at one high-volume hospital, was excluded, there was no difference between hybrid and cemented fixation in their direct comparative analysis, in keeping with our results.

We specifically examined the effects of age and sex on the revision risk in each cohort. Cemented fixation for older females may have had some advantage due to lower femoral bone density; however, we found no difference with hybrid fixation in females over 65 years, consistent with other authors (Nakama et al. 2012, Dalury 2016). Similarly, hybrid fixation may be of advantage to younger active males, but there was no difference between fixation types for males under 65 years.

We also assessed whether hybrid fixation lowered the infection risk given recent registry data suggesting a lower rate of revision for infection with certain TKR designs and bearing materials (Vertullo et al. 2017). Theoretically, a reduced burden of cement, cement particulate, and third-body wear could favorably alter the local immunomodulation of the joint environment (Spaan et al. 2013); however, our results suggest no advantage exists when only 1 major component is cemented.

In registry studies, the use of cross-linked polyethylene in TKR lowers the risk of loosening and lysis when compared with non-cross-linked polyethylene (de Steiger et al. 2015), presumably via a reduction in particle-related osteolysis. In our series, when the effect of XLPE was examined, it resulted in a 43% lower revision risk in the HP cohort compared with the CP cohort. It remains uncertain if this is due to an additive effect of XLPE when used with a lesser volume of cement, or some other unrecognized confounders such as patient selection or femoral component design.

When possible confounders were reduced using instrumented variable methodology, our analysis did not demonstrate superior survivorship with hybrid fixation overall. Consequently, it remains uncertain whether the extra cost of cementless femoral components is justified by the reduced operative time (Petursson et al. 2015) and possibly decreased bone loss given the excellent long-term results of cemented fixation of the femur.

By comparing revision risk based on surgeon fixation preference, we believe we have addressed concerns related to the potential for selection bias that may arise in a direct comparison of all cemented and hybrid TKR. To our knowledge this is the first time registry data have been used to investigate the outcome of surgeon preference in TKR fixation rather than directly comparing the long-term revision risk of hybrid and cemented TKR.

This study was specifically designed to address major confounders that may introduce bias between the hybrid and cemented fixation. The impact of potential differences due to age, gender, and primary diagnosis have been considered. In addition, prosthesis-specific factors such as the use of posterior cruciate stabilization, mobile bearing, patellar resurfacing, and cross-linked polyethylene were also considered. Only patients with osteoarthritis were included as other diagnoses such as rheumatoid arthritis are more likely to have a higher incidence of osteoporosis and consequently a higher use of cemented TKR. Mobile bearing TKR were excluded as they have a known higher revision risk compared with fixed bearing, and potentially could have a detrimental interaction with cementless femoral fixation (AOA National Joint Registry 2016). PS TKR were excluded as they have a higher revision risk than MS TKR and have a higher revision risk with hybrid fixation than cemented fixation (Vertullo et al. 2017).

As this is a registry analysis, some specific clarifications are important. First, a registry analysis differs from a clinical trial, in that while it can identify and monitor comparative national outcomes it cannot assign causality. Nonetheless, to optimize TKR survivorship, it is not vital to know why there is a difference between options, just that one exists, allowing all stakeholders to make shared informed decisions (Graves 2010). Second, another issue is unrecognized confounders. Unrecognized selection bias or confounding may have occurred, but, by focusing on the surgeon’s stability preference rather than the actual prosthesis used, this risk is minimized. Randomized controlled trials can reduce this selection bias; however, the current RCT literature showing no survivorship difference between hybrid and cemented fixation is underpowered to show a difference and has inadequate follow-up (Nakama et al. 2012). Surgeons with less surgical experience may prefer cemented fixation, but we restricted our analysis to surgeons who perform over 50 TKR per year to remove performance bias, as this has been previously cited as a large enough volume to exclude surgeon inexperience (Abdel et al. 2011). While the hybrid fixation preferring surgeons had on average more TKR per surgeon in the analysis than the cemented fixation preferring surgeons, the cemented fixation preferring surgeons performed more TKR per year on average and overall had performed a greater number of TKR. The AOANJRR collects level 1 and 2 data, hence comorbidities, patient-recorded outcome measures, and prosthesis alignment data are not collected. The AOANJRR only recently commenced recording ASA and BMI, and hence these factors could not be included in this analysis.

A possible limitation with any registry-based analysis is the data’s accuracy and validation. In the AOANJRR, after an initial capture rate of 96.8% (AOA National Joint Registry 2016), a sequential multi-level matching process against health department unit record data is undertaken, resulting in an almost complete dataset of primary and revision knee replacement in Australia.

In summary, there was no overall difference in the revision risk for the patients of surgeons who prefer hybrid fixation in minimally stabilized TKR, compared with the patients of surgeons who prefer cemented fixation. Only when the effects of alternative bearing surfaces were examined had the patients of surgeons who preferred hybrid fixation and utilized XLPE a 43% reduction in revision risk.

## Supplementary data

Table 4, Figure 4 and Figure 6 are available as supplementary data in the online version of this article, http://dx.doi.org/10.1080/17453674.2018.1449466

CJV designed the study, developed the methodology, performed the analysis, and wrote the manuscript. YP developed the methodology and performed the analysis. SEG and PLL collected the data, developed the methodology, performed the analysis, and wrote the manuscript.

The authors wish to thank Michelle Lorimer (BSc Hons) for technical assistance in the statistical methodology, the Australian Orthopaedic Association National Joint Replacement Registry, and the hospitals, orthopedic surgeons, and patients whose data made this work possible.

*Acta* thanks Gunnar Petursson and other anonymous reviewers for help with peer review of this study

## Supplementary Material

IORT_A_1449466_SUPP.PDF
