# Supplementary material for: The effect of surgeon’s preference for hybrid or cemented fixation on the long-term survivorship of total knee replacement: An analysis of 39,623 prostheses from the Australian Orthopaedic Association National Joint Replacement Registry
Source: Acta Orthop. 2018 Mar 12;89(3):329–35. doi: 10.1080/17453674.2018.1449466 (PMC6055785; doi:10.1080/17453674.2018.1449466)
Supplement: IORT_A_1449466_SUPP.PDF [file IORT_A_1449466_SM3659.pdf]

Supplementary data

Table 4. Revision diagnosis of primary total knee replacement by surgeon preference with osteoarthritis

Revision diagnosis	Routinely cemented (n = 30,544) n	%	% of revisions	Routinely hybrid (n = 9,079) n	%	% of revisions
Infection	167	0.5	28.7	48	0.5	30.0
Loosening/lysis	101	0.3	17.4	23	0.3	14.4
Patellofemoral pain	70	0.2	12.0	23	0.3	14.4
Pain	56	0.2	9.6	13	0.1	8.1
Instability	46	0.2	7.9	19	0.2	11.9
Other	142	0.5	24.4	34	0.4	21.2
Number of revisions	582	1.9	100	160	1.8	100

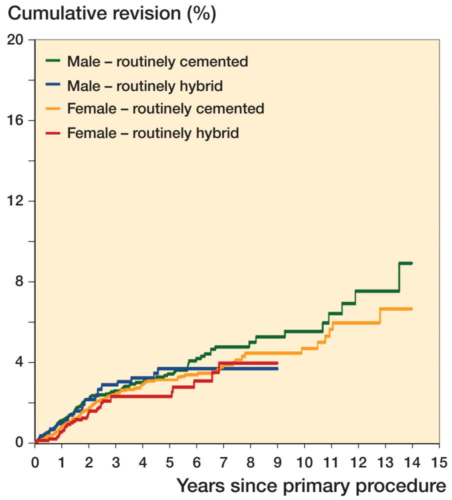

Figure 4. Cumulative percentage revision of primary total knee replacement for patients aged < 65 years by sex and surgeon fixation preference in patients with osteoarthritis.
HR adjusted for age, entire period:
Male RC vs male RH: HR = 1.03 (0.70–1.50), p=0.9)
Male RH vs female RH: HR = 1.32 (0.82–2.12), p = 0.3)
Male RC vs female RC: HR = 1.18 (0.93–1.50), p = 0.2)
Female RC vs female RH: HR = 1.15 (0.79–1.67), p = 0.5)

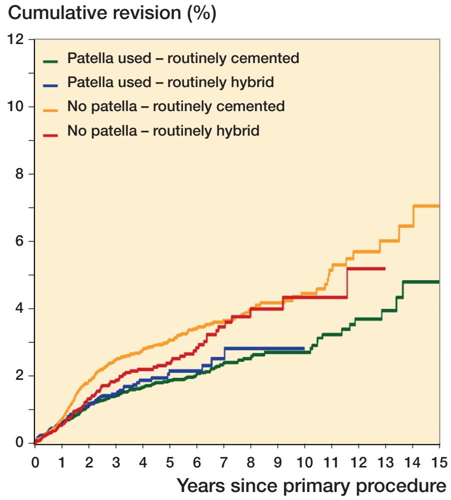

Figure 6. Cumulative percentage revision of primary total knee replacement by patella usage and surgeon fixation preference in patients with osteoarthritis.
HR adjusted for age and sex, entire period:
Patella used RC vs patella used RH: HR = 0.92 (0.71–1.21), p = 0.6
Patella used RH vs no patella RH: HR = 0.83 (0.60–1.13), p = 0.2
Patella used RC vs No patella RC: HR = 0.67 (0.56–0.78), p < 0.001
No patella RC vs no patella RH: HR = 1.15 (0.91–1.44), p = 0.3
